# Squamous Metaplasia of Lactiferous Ducts (SMOLD) in a Male Patient: Clinical, Dermoscopic, and Histopathological Insights

**DOI:** 10.3390/diagnostics15192489

**Published:** 2025-09-29

**Authors:** Beata Zagórska, Przemysław Miłosz, Jakub Żółkiewicz, Urszula Maińska, Martyna Sławińska

**Affiliations:** 1 Department and Clinic of Dermatology, Venerology and Allergology, Medical University of Gdansk, Mariana Smoluchowskiego 17, 80-214 Gdańsk, Poland; beatazagorska@gumed.edu.pl (B.Z.); ulakobus@gumed.edu.pl (U.M.); 2 Department of Pathomorphology, Medical University of Gdansk, Mariana Smoluchowskiego 17, 80-214 Gdańsk, Poland; przemyslaw.milosz@gumed.edu.pl

**Keywords:** SMOLD, squamous metaplasia of lactiferous ducts, Zuzka’s disease, male case, dermoscopy

## Abstract

We present the case of a 44-year-old male patient who presented to a dermatology outpatient clinic due to an asymmetric swelling of the left nipple. The patient reported a burning sensation within the area, persisting for approximately six months. Due to the inconclusive dermoscopic findings and lack of improvement following empirical treatment, a biopsy was performed. Histopathological examination revealed keratinizing squamous metaplasia of the lactiferous ducts (SMOLD).

**Figure 1:**
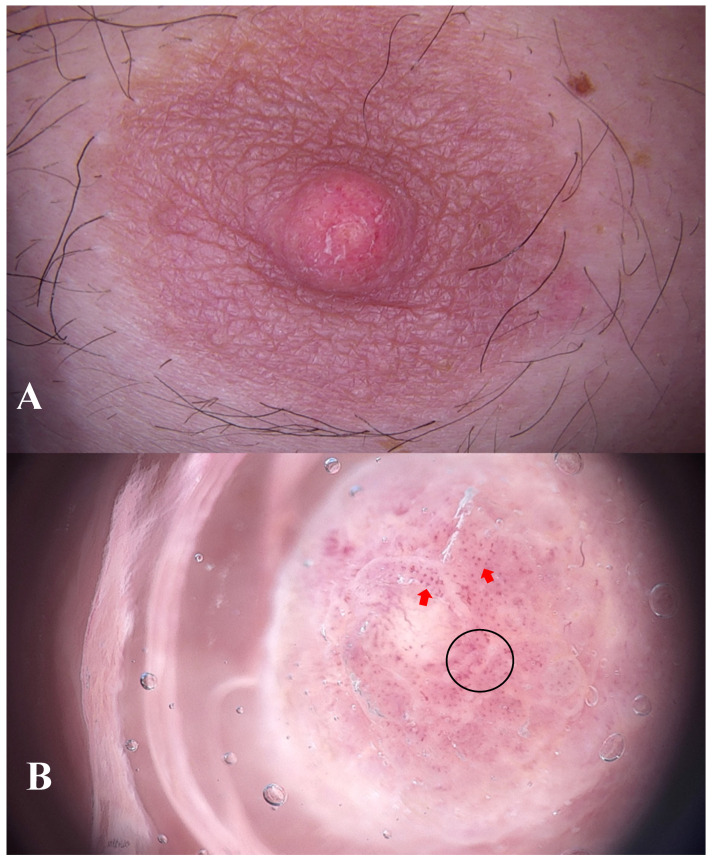
A 44-year-old male non-smoker presented to the dermatology outpatient clinic with asymmetric swelling of the left nipple and subtle scaling (**A**). He was otherwise healthy, with no history of hormonal disorders. There was no family history of breast pathology. The lesion had been present for six months and was accompanied by a burning sensation. The patient associated the onset of the symptoms with mechanical trauma experienced during wrestling training. No prior treatment had been given. Ultrasound examination of the breast showed no abnormalities. Further imaging (mammography or MRI) was not pursued, as neither clinical findings nor patient history suggested malignancy. Dermoscopy revealed polymorphic (black circle) and dotted vessels (red arrows) features suggestive of increased vascularity associated with neoplastic or inflammatory processes (**B**), FotoFinder Vexia Medicam 800 HD; 20× magnification; non-polarized light; immersion: ultrasound gel). Due to the inconclusive clinical and dermoscopic findings, empirical topical treatment with fusidic acid and betamethasone was initiated.

**Figure 2:**
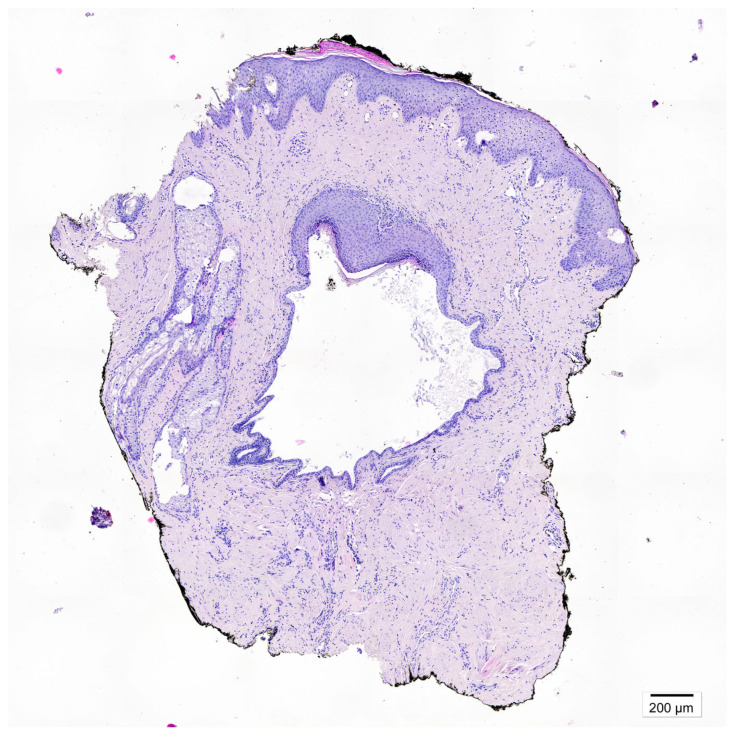
After three weeks, no clinical improvement was observed. The patient was referred for a diagnostic punch biopsy, performed 4 weeks after discontinuation of treatment. A breast skin fragment containing a cystically dilated lactiferous duct was obtained. Microscopic examination revealed squamous metaplasia extending beyond the normal transition point of the ductal epithelium, with keratin-filled ducts and rupture-associated inflammatory reaction containing multinucleated giant cells. Given the lesion’s asymptomatic presentation and the patient’s preference, surgical excision was not performed. Instead, careful clinical follow-up in the dermatology outpatient clinic was implemented. After four months, the patient remains free of recurrent symptoms. Squamous metaplasia of lactiferous ducts (SMOLD)**,** previously also known as Zuska’s disease is a rare, benign breast disorder characterized by keratinizing squamous metaplasia of the lactiferous ducts [1]. Epidemiology of the disease is poorly defined [2]. The condition is most frequently reported in middle-aged women and rarely observed in men [3]. Smoking, including secondhand exposure is the main risk factor. Nicotine exerts either a direct or indirect cytotoxic influence on the epithelial cells lining the subareolar milk ducts. Our patient had no history of active or passive smoking. Less common associations include nipple piercing and congenital cleft nipple [4]. Mechanical trauma has not been reported as a recognized risk factor. In our patient, symptom onset followed a localized injury. Notably, nipple piercing—another documented risk factor—can also be considered a form of mechanical insult. This highlights that epithelial disruptions have been observed in some cases. Clinically, SMOLD exhibits variable presentations and may mimic inflammatory breast carcinoma, complicating differential diagnosis [3]. Typical cases occur in middle-aged women and are recurrent and painful, whereas atypical cases, particularly in men, may be minimally symptomatic or asymptomatic. Some reports describe unilateral involvement, others bilateral. Male patients more often present with subtle or atypical symptoms, highlighting the need for careful clinical assessment [2,5]. Its nonspecific manifestation often leads to misdiagnosis and unnecessary interventions [6]. Imaging, including ultrasound and mammography, may reveal ductal dilatation or periductal changes, but these findings are not pathognomonic [5]. Ultrasound plays a critical role in evaluation, particularly in male patients [2,7]. At the time of writing this manuscript, we have not found any reports in the literature regarding the dermoscopic features of SMOLD. Dermatoscopy is increasingly regarded as an indispensable diagnostic tool in dermatological practice. Characteristic vascular patterns have been reported in nipple disorders, including nipple eczema (punctate or linear vessels over an erythematous background), Paget’s disease (irregular linear or serpentine vessels), and ductal carcinoma in situ (atypical linear or branching vessels with irregular distribution) [8,9,10]. In the present case, we identified polymorphic and punctate vessels. Although such features have not previously been described in the context of SMOLD, they may represent a potential (however non-characteristic) diagnostic clue. Microscopically, SMOLD is defined by cystically dilated lactiferous ducts lined by keratinizing squamous epithelium, often accompanied by periductal inflammation or a granulomatous reaction [4]. Standard treatment involves surgical excision, especially in symptomatic cases or when malignancy cannot be excluded [6]. This case provides several clinical insights. SMOLD should be considered in the differential diagnosis of subareolar masses in both sexes. Dermoscopy can offer additional information on vascular patterns, but histopathology remains the diagnostic gold standard. In asymptomatic patients, careful observation may be preferred over immediate surgical intervention. Although benign, SMOLD’s clinical and radiological resemblance to DCIS or invasive carcinoma necessitates careful evaluation [5]. Awareness among dermatologists, breast surgeons, and pathologists is crucial to prevent overtreatment [11].

## Data Availability

All relevant data are within the manuscript.

## References

[1] Zuska J.J., Crile G., Ayres W.W. (1951). Fistulas of lactifierous ducts. Am. J. Surg..

[2] Gkionis I.G., Giakoumakis M.I., Liva D., Tsioulos G., Matalliotakis M., Vrontaki M., Giakoumakis K.I., Cavallo G., Laliotis A. (2023). Zuska’s disease in a male patient. The critical role of ultrasound imaging in diagnosis and management of this rare entity. Radiol. Case Rep..

[3] Ofri A., Dona E., O’Toole S. (2020). Squamous metaplasia of lactiferous ducts (SMOLD): An under-recognised entity. BMJ Case Rep..

[4] Serrano L.F., Rojas-Rojas M.M., Machado F.A. (2020). Zuska’s breast disease: Breast imaging findings and histopathologic overview. Indian J. Radiol. Imaging.

[5] Ravichandran S. Zuska Disease: The Role of Histopathology in this Underdiagnosed Breast Entity. Proceedings of the ASDP 59th Annual Meeting, Chicago, IL, USA, 17–23 October 2022.

[6] Szczudlo-Chrascina J., Bojar P., Holecki M., Niewiadomska A., Steinhof-Radwanska K. (2025). The inflammatory breast cancer mimicker—The SMOLD syndrome—Rare complication of smoking. Ginekol. Pol..

[7] Johnson S.P., Kaoutzanis C., Schaub G.A. (2014). Male Zuska’s disease. BMJ Case Rep..

[8] Wu C., Jia Q.N., Fang K., Zeng Y.P. (2023). Skin diseases of the nipple and areola complex: A case series study from China. Front. Med..

[9] Apalla Z., Errichetti E., Kyrgidis A., Stolz W., Puig S., Malvehy J., Zalaudek I., Moscarella E., Longo C., Blum A. (2019). Dermoscopic features of mammary Paget’s disease: A retrospective case-control study by the International Dermoscopy Society. J. Eur. Acad. Dermatol. Venereol..

[10] Alfaro R.Q., Siripunvarapon A.H., Quinio M.F.S., Duran C.M.H., Pamin J.R., Habito C.M.R. (2024). A dermoscopy case series of cutaneous metastases from breast cancer in Filipino females. JEADV Clin. Pract..

[11] D’Alfonso T.M., Ginter P.S., Shin S.J. (2015). A Review of Inflammatory Processes of the Breast with a Focus on Diagnosis in Core Biopsy Samples. J. Pathol. Transl. Med..

